# Understanding HPV Vaccine Initiation and Intention Among Central American Immigrant Parents in the United States: The Role of Vaccine Literacy and Healthcare Provider Recommendations

**DOI:** 10.3390/vaccines13020130

**Published:** 2025-01-27

**Authors:** Doris Lucero, Virginia A. Moreno, Denisse Delgado, Axel Hernandez Nieto, Nachalie Rodriguez-Cruz, Qun Le, Ana Cristina Lindsay

**Affiliations:** 1 Department of Biology, College of Sciences and Mathematics, University of Massachusetts Boston, Boston, MA 02125, USA; doris.lucero001@umb.edu (D.L.); a.hernandeznieto001@umb.edu (A.H.N.); 2 Department of Exercise and Health Sciences, Robert J and Donna Manning College of Nursing and Health Sciences, University of Massachusetts Boston, Boston, MA 02125, USA; virginia.arango001@umb.edu (V.A.M.); n.rodriguezcruz001@umb.edu (N.R.-C.); 3 McCormack School of Public Policy, University of Massachusetts Boston, Boston, MA 02125, USA; d.delgadovazquez@umb.edu; 4 Department of Public Health, Zuckerberg College of Health Sciences, University of Massachusetts Lowell, Lowell, MA 01854, USA; qun.le@uml.edu; 5 Department of Urban Public Health, Robert J and Donna Manning College of Nursing and Health Sciences, University of Massachusetts Boston, Boston, MA 02125, USA

**Keywords:** HPV vaccine initiation, vaccine intention, HPV vaccine literacy, Central American immigrants, healthcare provider recommendation

## Abstract

**Background/Objectives**: The HPV vaccine is key to preventing HPV-related cancers, yet vaccination rates are low, particularly among immigrant and ethnic minority groups. This study explored factors influencing HPV vaccine initiation and intention among Central American immigrant parents in the U.S. **Methods**: A cross-sectional study with parents of children aged 11 to 17 years. **Results**: Among the 168 parents (53.8% mothers, 46.2% fathers) in this study, 20% reported that their children had initiated the HPV vaccine and 23% of parents of unvaccinated children intended to vaccinate within the next 12 months. Sociodemographic factors, including the parent’s gender and length of U.S. residence, were significant predictors of HPV vaccine initiation and intention. Higher vaccine literacy was also a key factor, with parents with higher vaccine literacy scores being more likely to initiate vaccination and express intent to vaccinate their children. HCP communication strongly predicted both vaccine initiation and intention, yet fewer than 30% of parents received HPV vaccine information from a HCP, and less than 28% received a vaccine recommendation. In the multiple logistic regression analysis, receiving HPV vaccine information from a HCP was the strongest predictor of vaccine initiation (AOR = 93.23, 95% CI = 14.50–599.63, *p* < 0.001), adjusting for other variables. For vaccination intention, significant predictors included the length of U.S. residence (AOR = 0.84, 95% CI = 0.75–0.95, *p* < 0.01), having a U.S.-born child (AOR = 10.47, 95% CI = 1.51–72.68, *p* < 0.05), and receiving vaccine recommendation from a HCP (AOR = 14.73, 95% CI = 1.77–122.32, *p* < 0.05). **Conclusions**: To improve vaccination rates, interventions should enhance HCP training, strengthen provider–patient communication, and address vaccine literacy through culturally tailored strategies and community engagement.

## 1. Introduction

Human papillomavirus (HPV) is one of the most prevalent sexually transmitted infections globally and a significant public health challenge due to its association with various cancers, including cervical, oropharyngeal, anal, vulvar, vaginal, and penile cancers [1,2]. Although the HPV vaccine is a proven and effective strategy for preventing these cancers, vaccine uptake remains suboptimal, particularly among immigrant communities [3,4].

Vaccine literacy plays a crucial role in shaping parents’ decisions regarding the HPV vaccination for their children [5,6,7,8]. Understanding the benefits, risks, and necessity of the HPV vaccine—the core components of vaccine literacy—is essential for promoting informed vaccination decisions and increasing vaccination rates [9,10,11]. Research has shown that parents with higher vaccine literacy are more likely to recognize the importance of the HPV vaccine, leading to a greater intention to vaccinate [6,8,12]. In contrast, gaps in HPV vaccine knowledge can discourage parents from seeking vaccination for their children [9,13,14]. Cultural beliefs also significantly influence vaccine perceptions and healthcare decisions, often contributing to HPV vaccine hesitancy [14,15,16,17,18,19]. Understanding how cultural factors intersect with vaccine literacy is essential for developing communication strategies that effectively resonate with culturally diverse immigrant populations [19].

Another key factor in promoting HPV vaccination is the critical role healthcare providers (HCPs) play in influencing parental decisions about their children’s health [5,20,21,22,23]. Previous research has shown that strong recommendations from HCPs can significantly boost vaccine initiation and intent, particularly among populations facing unique barriers, such as immigrants [5,24,25,26]. Clear and supportive guidance helps parents understand the importance of the HPV vaccine and its potential to prevent certain cancers [27]. This support is particularly vital for immigrant parents, who may face challenges, such as low literacy, logistical barriers, or language difficulties when navigating healthcare systems [14,28].

Central American immigrants, especially those from the Northern Triangle countries of El Salvador, Guatemala, and Honduras, represent a growing and significant demographic in the United States (U.S.) [5,6]. However, comprehensive research on HPV vaccine uptake among Central American immigrant parents in the U.S. is limited, creating a critical gap in understanding how to effectively address their needs [7,8,9]. Understanding the factors influencing vaccination behaviors, such as HPV vaccine uptake and intention, among these populations is crucial for developing targeted interventions. This knowledge is essential for improving vaccine uptake, reducing HPV-associated cancers, and addressing public health disparities.

Like other ethnically minoritized populations, Central American immigrant parents face several challenges that impact their vaccine behaviors, including barriers to HPV vaccination for their children [11,12]. These challenges include socioeconomic factors, low educational attainment, limited healthcare access, cultural beliefs, language barriers, and varying levels of health literacy [8,9,10,11,12,13]. As prior research has suggested, these barriers can hinder parents’ understanding of medical terminology, their ability to navigate healthcare systems, and their access to reliable health information, factors that are critical for making informed vaccination decisions [8,9,10,11,12,13]. Given the limited research on HPV vaccination behaviors among Central American immigrants in the U.S., this study was designed to examine the factors influencing both HPV vaccine initiation and intention among parents in these communities.

## 2. Materials and Methods

This study is part of a larger mixed methods research project conducted across the United States to explore and understand the factors influencing HPV vaccine uptake among Central American parents from El Salvador, Guatemala, and Honduras, collectively known as the Northern Triangle countries [6]. The research was guided by the Social Ecological Model (SEM) [29] and the Health Belief Model (HBM) [30]. Data were collected in several states with significant Central American immigrant populations, including California, Florida, Texas, New York, New Jersey, Illinois, Maryland, Virginia, Arizona, and Massachusetts, regions where access to preventive healthcare services and vaccine information may vary.

### 2.1. Sample and Setting

The present study used a cross-sectional design, with a convenience sample of Central American parents from El Salvador, Guatemala, and Honduras residing in the U.S. The inclusion criteria were as follows: (1) self-identification as Salvadoran, Guatemalan, or Honduran; (2) having at least one child aged 11 to 17 years; (3) being 21 years of age or older; (4) residing in the U.S. for at least six months; (5) proficiency in Spanish or English; and (6) the ability to provide informed consent.

### 2.2. Recruitment

Recruitment efforts focused on community outreach, utilizing both traditional methods and digital platforms. Participants were recruited through community organizations, social media platforms, local events, and network sampling, where enrolled participants referred others. Bilingual study staff conducted screenings to ensure eligibility. These recruitment methods were successfully implemented in our previous studies to engage immigrant populations, including Latino parents, providing a strong foundation for participant enrollment [31,32,33,34].

### 2.3. Data Collection and Survey Measures

Prior to the start of the survey interview, all participants were informed in their preferred language (Spanish or English) about the study’s purpose, procedures, and their rights, including the right to withdraw at any time without penalty. Informed consent was obtained electronically before proceeding with the interviewer-administered questionnaire.

The data were collected via structured telephone interviews, using an online questionnaire via Qualtrics, from December 2022 to May 2023. Trained bilingual interviewers conducted the surveys, ensuring cultural competency and adherence to the study’s protocols. All interviewers received training on the survey content, interviewing techniques, and cultural sensitivity to ensure accurate and respectful data collection. The participants received a USD $40 gift card as compensation for their time and participation.

The 164-item survey, designed to assess the participants’ knowledge, attitudes, and behaviors regarding HPV and the HPV vaccine, was developed based on the existing literature, validated tools, and guided by the SEM [29] and the HBM [30]. The present study focused on the following variables:

#### 2.3.1. HPV Vaccine Initiation and Intention

The primary outcome variables were HPV vaccine initiation and intention to vaccinate among parents who had not yet initiated vaccination for their oldest child. To assess whether parents had initiated the vaccine series for their 11–17-year-old child (vaccine initiation), parents were asked, “Has your child received at least one dose of the HPV vaccine?” (response options: yes, no, don’t know). Parents who had not initiated the HPV vaccine, or who were unsure, were asked about their intention to vaccinate within the next year (vaccine intention). The question was, “How likely are you to have your child receive the HPV vaccine within the next 12 months?”, with response options based on a 5-point Likert scale, ranging from “extremely likely” to “not likely at all”. These questions have been previously used in studies with Latino and immigrant populations in the U.S. [35,36,37,38,39,40,41,42,43].

#### 2.3.2. Healthcare Provider Recommendations and Information Sources

The HCP recommendation was assessed by asking parents, “Has a healthcare provider ever recommended the HPV vaccine to your age-eligible child?” (response options: yes, no, don’t know). The sources of information about the HPV vaccine were assessed by asking, “How did you hear about the HPV vaccine?” Parents could select all sources from which they had obtained information. The response options included family members, friends, HCPs, school, TV, radio, newspapers, the internet, magazines, public health campaigns, and other sources. Multiple responses were allowed. These questions have been used in studies involving parents, including Latino populations, in the U.S. [35,39,40].

#### 2.3.3. HPV Vaccine Literacy

HPV vaccine knowledge was assessed by asking parents about their awareness of the HPV vaccine, its benefits, the recommended vaccination age, and safety. The response options were coded as true/false/don’t know. For analysis, these items were treated as dichotomous measures (correct = 1, incorrect = 0). The total number of correct responses was calculated and divided by the total number of questions (12), yielding a score ranging from 0 to 100%. The sum of correct responses was presented as a continuous variable, with higher scores indicating greater HPV vaccine literacy. The scores ranged from 0 to 12 based on correct answers. These questions have been previously used in studies with Latino populations in the U.S. [37,42,44,45].

#### 2.3.4. Demographic and Acculturation Characteristics

The demographic data collected from the participants included age, gender, marital status, education, income, employment status, health insurance, parents’ country of birth, primary language, length of U.S. residence, and acculturation level. These questions have been previously used in studies involving Latino populations, including Central American parents, and have also been employed in research conducted by our team [31,32,33,34].

Acculturation was assessed using the Short Acculturation Scale for Hispanics (SASH) [46]. The SASH is a 12-item scale that measures language use, media preferences, and ethnic social relationships, and has demonstrated strong reliability (Cronbach’s alpha: 0.72–0.92) [46,47]. The acculturation scores were calculated by averaging the responses across all 12 items, with scores ranging from 1 to 5. A score of 2.99 or higher was classified as indicating higher acculturation [46].

### 2.4. Data Analysis

Descriptive statistics (mean, standard deviation, frequencies, percentages) summarized the sociodemographic characteristics and HPV-related variables. Inferential statistics, including chi-square tests, Fisher’s exact test, and the Wilcoxon Rank Sum test, explored associations between the demographic variables, HPV vaccine literacy, vaccine initiation, and intention to vaccinate within the next 12 months. The responses to vaccine intention, coded based on a 5-point Likert scale, were dichotomized into “extremely likely/very likely/likely” and “not too likely/not likely at all/unsure”. Bivariate and multiple logistic regression models were used to evaluate the factors associated with vaccine initiation and intention to vaccinate within the next 12 months, separately. The results are presented, along with odds ratio (OR) values, within 95% CIs. Probability (*p*) values < 0.05 were used to indicate statistical significance. All the analyses were conducted with SAS 9.4. [48].

### 2.5. Ethical Considerations

This study was conducted in accordance with the ethical principles outlined in the Declaration of Helsinki, which provides ethical guidelines for medical research involving human participants [49]. All procedures involving human participants were approved by the University of Massachusetts Boston under protocol number [#3036 2022047, approval date 10 March 2022].

Informed consent was obtained from all the participants prior to their inclusion in the study. Consent was obtained in the participants’ preferred language (Spanish or English) to ensure that they had a clear understanding of the information provided. The participants were provided with detailed information about the study’s purpose, procedures, potential risks, and benefits, and were assured that participation was voluntary and that they could withdraw at any time without penalty. To protect participant confidentiality, all the data were anonymized and stored securely, accessible only to the research team.

## 3. Results

### 3.1. Sociodemographic and Cultural Characteristics of the Sample

Table 1 presents the sociodemographic characteristics of the study sample (N = 168), comprising 53.8% mothers (mean age = 41.6 years) and 46.2% fathers (mean age = 49.3 years, *p* < 0.001). Most parents (75.0%) were married or living with a partner, with more fathers being married or living with a partner (84.8%) than mothers (66.3%, *p* = 0.006). Educational attainment was similar across genders, with 51.2% having less than a high school education (*p* = 0.27). Employment rates were high (85.7%), but more fathers (94.9%) were employed compared to mothers (77.5%, *p* = 0.002). Significant income differences were noted, with 38.1% earning below USD $50,000, more so among mothers (55.1%) than fathers (19.0%, *p* < 0.001). Most had health insurance (87.5%). Health insurance coverage was slightly higher among mothers (92.1%) than fathers (82.3%, *p* = 0.05).

Regarding the parents’ ethnicity, 36.9% of parents identified as Salvadoran, 32.1% as Guatemalan, and 31.0% as Honduran, with no significant ethnic differences between mothers and fathers (*p* = 0.43). The parents had lived in the U.S. for an average of 16.9 years, with fathers living in the country longer (mean = 18.6 years) compared to mothers (mean = 15.4 years, *p* = 0.03). Spanish was the primary language spoken at home by 87% of the sample, more commonly among mothers (89.9%) than fathers (83.5%, *p* = 0.02). Nearly all parents (94.6%) reported low acculturation levels (SASH score < 2.99), with no significant gender differences observed (*p* = 0.50).

On average, the parents had 1.2 children aged 11–17, with a mean age of 14.2 years for the index child (oldest child between 11–17 years) and no significant age difference (*p* = 0.60). Sixty-three percent of the index children were U.S. born, 59% were male (*p* = 0.003), and nearly all had a regular HCP (97.0%).

**Table 1:** Sociodemographic and cultural characteristics of the sample (N = 168).

Variables	Total Sample N = 168 (%)	Mothers N = 89 (%)	Fathers N = 79 (%)	*p*-Value
**Age (Mean, SD)**	45.3 (10.1)	41.6 (7.7)	49.3 (11.0)	<0.001 ***
**Marital status**				
Married/living with partner	126 (75.0%)	59 (66.3%)	67 (84.8%)	0.006 **
Other	42 (25.0%)	30 (33.7%)	12 (15.2%)	
**Educational level**				
<High school	86 (51.2%)	42 (47.2%)	44 (55.7%)	0.27
≥High school	82 (48.8%)	47 (52.8%)	35 (44.3%)	
**Household income**				
<USD $50,000	64 (38.1%)	49 (55.1%)	15 (19.0%)	<0.001 ***
≥USD $50,000	95 (56.5%)	33 (37.1%)	62 (78.5%)	
Missing	9 (5.4%)	7 (7.8%)	2 (2.5%)	
**Employment status**				
Yes	144 (85.7%)	69 (77.5%)	75 (94.9%)	0.002 **
No	24 (14.3%)	20 (22.5%)	4 (5.1%)	
**Health insurance status**				
Yes	147 (87.5%)	82 (92.1%)	65 (82.3%)	0.05 *
No	21 (12.5%)	7 (7.9%)	14 (17.7%)	
**Parent’s ethnicity**				
Salvadoran	62 (36.9%)	37 (40.6%)	25 (31.7%)	0.41
Honduran	52 (31.0%)	26 (29.2%)	26 (32.9%)	
Guatemalan	54 (32.1%)	26 (29.2%)	28 (35.4%)	
**Primary language spoken at home**				
English	6 (3.6%)	5 (5.6%)	1 (1.3%)	0.02 *
Spanish	146 (86.9%)	80 (89.9%)	66 (83.5%)	
Both	16 (9.5%)	4 (4.5%)	12 (15.2%)	
**Parent length of residence in the U.S.**	16.9 (9.6)	15.4 (9.3)	18.6 (9.8)	0.03 *
**Parent’s acculturation level**				
<2.99	159 (94.6%)	83 (93.3%)	76 (96.2%)	0.50
≥2.99	9 (5.4%)	6 (6.7%)	3 (3.8%)	
**Total number of children 11–17 years**	1.2 (0.5)	1.2 (0.5)	1.2 (0.5)	0.90
**Age of index child (11–17 years)**				
11	27 (16.1%)	14 (15.7%)	13 (16.5%)	0.51
12	26 (15.5%)	11 (12.4%)	15 (19.0%)	
13	14 (8.3%)	10 (11.2%)	4 (5.1%)	
14	19 (11.3%)	9 (10.1%)	10 (12.7%)	
15	23 (13.7%)	11 (12.4%)	12 (15.2%)	
16	26 (15.5%)	17 (19.1%)	9 (11.4%)	
17	33 (19.6%)	17 (19.1%)	16 (20.3%)	
**Mean, SD**	14.2 (2.2)	14.2 (2.1)	14.1 (2.2)	0.60
**Sex of the index child**				
Male	99 (58.9%)	43 (48.3%)	56 (70.9%)	0.003 **
Female	69 (41.1%)	46 (51.7%)	23 (29.1%)	
**Index child birth place**				
Foreign born	62 (36.9%)	34 (38.2%)	28 (35.4%)	0.71
U.S. born	106 (63.1%)	55 (61.8%)	51 (64.6%)	
**Index child has regular HCP**				
Yes	163 (97.0%)	87 (97.8%)	76 (96.2%)	0.67
No	5 (3.0%)	2 (2.2%)	3 (3.8%)	

Note: HCP = healthcare provider; *p* < 0.05 *, *p* < 0.01 **, *p* < 0.001 ***.

### 3.2. HPV Vaccine Initiation and Intent to Vaccinate Among Parents of Unvaccinated Children

As shown in Table 2, less than 28% of the parents reported that their child’s HCP had recommended the HPV vaccine. A significantly higher proportion of mothers (39.3%) reported that their child’s HCP had recommended the HPV vaccine compared to fathers (13.9%, *p* < 0.001). Additionally, only about 20% of parents reported that their child had initiated the HPV vaccination (at least one dose of the vaccine). Mothers (27.0%) were more likely to report that their child had initiated the HPV vaccination than fathers (12.7%) (*p* = 0.02).

Among the parents (n = 134) whose children had not yet initiated the HPV vaccination, only 23.1% expressed that they were likely to vaccinate their child within the next 12 months. Mothers (32.3%) were significantly more likely to report an intent to vaccinate their child in the next year compared to fathers (14.5%) (*p* = 0.02).

Regarding sources of information, a HCP was the most common source of information about the HPV vaccine, with about 30% of parents reporting having received information from a HCP (doctor or nurse), with a significant difference between mothers (43.8%) and fathers (14.1%) (*p* < 0.001). Other sources of information included friends (16.8%), public health campaigns (12%), TV (12%), the internet (11.4%), and family members (10.8%). However, none of these sources showed a significant gender difference.

**Table 2:** HPV vaccine initiation, intention to vaccinate within the next 12 months, and sources of information (N = 168).

Variables	Total Sample N = 168 (%)	Mothers N = 89 (%)	Fathers N = 79 (%)	*X* ^2^	*p*-Value
**HCP recommended the HPV vaccine**					
Yes	46 (27.4%)	35 (39.3%)	11 (13.9%)	13.58	<0.001 ***
No/Don’t know	122 (72.6%)	54 (60.7%)	68 (86.1%)		
**Vaccine initiation (receipt of at least one dose of the HPV vaccine)**					
Yes	34 (20.2%)	24 (27.0%)	10 (12.7%)	5.308	0.02 *
No/Don’t know	134 (79.8%)	65 (73.0%)	69 (87.3)		
**Intention to vaccinate in the next 12 months among unvaccinated (*n* = 134)**					
Likely/Very likely/Extremely likely	31 (23.1%)	21 (32.3%)	10 (14.5%)	7.719	0.02 *
Not too likely/Not likely at all	38 (28.4%)	13 (20.0%)	25 (36.2%)		
Unsure/Don’t know	65 (48.5%)	31 (47.7%)	34 (49.3%)		
**HPV vaccine information sources**					
HCP (doctor or nurse)	50 (29.9%)	39 (43.8%)	11 (14.1%)	17.502	<0.001 ***
Family members	18 (10.8%)	8 (9.0%)	10 (12.8%)	0.635	0.43
Internet	19 (11.4%)	10 (11.2%)	9 (11.5%)	0.004	0.95
Friends	28 (16.8%)	16 (18.0%)	12 (15.4%)	0.200	0.65
TV	20 (12.0%)	10 (11.2%)	10 (12.8%)	0.099	0.75
Public Health Campaigns	20 (12.0%)	11 (12.4%)	9 (11.5%)	0.027	0.87

*p* < 0.05 *, *p* < 0.001 ***.

### 3.3. HPV Vaccine Literacy

The results of the descriptive analysis of HPV vaccine literacy are presented in Table 3. On average, parents correctly answered 50% of the HPV vaccine literacy questions. Notably, 73.2% of parents correctly identified that the statement about the HPV vaccine and Pap test was false. Additionally, 63.7% of parents recognized that the HPV vaccine does not offer protection against all sexually transmitted infections, 57.7% correctly answered that the HPV vaccine offers protection against genital warts, and 60.1% knew that the HPV vaccine prevents new infections but does not treat existing ones.

Approximately 50% of parents correctly answered that the HPV vaccine is recommended for routine vaccination at age 11 or 12 years, with the option to start at age 9, and that someone who has had the HPV vaccine can still develop cervical cancer. Fewer than half of the parents answered the remaining items correctly.

Parents whose children had initiated the HPV vaccine were significantly more likely to correctly answer HPV vaccine literacy items, with 70.6% correctly answering that the vaccine requires at least two doses, compared to 30.6% of those whose children had not initiated vaccination (*p* < 0.001). Similarly, 64.7% of parents whose children had initiated vaccination correctly answered that the vaccine is given as a series of two or three doses, depending on the child’s age at initial vaccination, compared to 24.6% of those whose children had not (*p* < 0.001). Additionally, 82.4% of parents whose children had initiated vaccination correctly answered that the HPV vaccine is recommended for routine vaccination at age 11 or 12, with vaccination starting at age 9, compared to 41.0% of those whose children had not (*p* < 0.001). Parents whose children had initiated vaccination were also more likely to correctly identify that the HPV vaccine does not cure HPV (61.8% vs. 38.8%, *p* = 0.02). Furthermore, parents who reported an intention to vaccinate their children within the next 12 months were more likely to correctly answer that the vaccine requires at least two doses (45.2% vs. 26.2%, *p* = 0.04).

An HPV vaccine literacy score, calculated by adding up the total correct answers and dividing by the total number of questions, was significantly higher among parents whose children had initiated HPV vaccination (0.66) compared to those who had not (0.46, *p* < 0.001). Furthermore, parents who intended to vaccinate their children in the next 12 months had higher HPV literacy scores (0.52) compared to those who did not report such intentions (0.44, *p* = 0.05).

### 3.4. Bivariate and Multiple Logistic Regression

Table 4 and Table 5 present the results of the bivariate and multiple logistic regression analyses, examining the association between various predisposing, enabling, and need factors and HPV vaccine initiation and intention to vaccinate within the next 12 months, respectively. The analyses revealed several significant findings.

#### 3.4.1. Vaccine Initiation

Bivariate analyses revealed several significant associations between predisposing, enabling, and need factors and HPV vaccine initiation (Table 4). In terms of the predisposing factors, some parental and child characteristics were associated with vaccine initiation. The parent’s gender was significantly associated with vaccine initiation, with mothers being more likely than fathers to initiate the vaccine (OR = 2.55, *p* = 0.02). Additionally, the length of residence in the U.S. was positively associated with vaccine initiation, with parents living longer in the U.S. having higher odds of initiating vaccination (OR = 1.05, *p* = 0.02). Other factors, such as the parent’s age, marital status, ethnicity, and the primary language spoken at home showed no statistically significant associations with vaccine initiation.

Regarding the child’s characteristics, only the country of birth was significantly associated with vaccine initiation. Children born in the U.S. were significantly more likely to have received the vaccine compared to those born outside the U.S. (OR = 3.35, *p* = 0.01). None of the other factors, such as the child’s sex or age, were significantly associated with vaccine initiation.

**Table 3:** Descriptive analysis of HPV vaccine literacy (N = 168).

HPV Vaccine Literacy (12 Items)	N (%)	HPV Vaccine Initiation (N = 168)			HPV Vaccination Intention Within the Next 12 Months (n = 134)		
		N (%)		*p*-Value	N (%)		*p*-Value
		No	Yes		No	Yes	
The HPV vaccine requires at least two doses. (T)	65 (38.7%)	41 (30.6%)	24 (70.6%)	<0.001 ***	27 (26.2%)	14 (45.2%)	0.04 *
The HPV vaccine offers protection against all sexually transmitted infections. (F)	107 (63.7%)	85 (63.4%)	22 (64.7%)	0.89	68 (66.0%)	17 (54.8%)	0.26
The HPV vaccine is recommended for routine vaccination at age 11 or 12 years. Vaccination can be started at age 9. (T)	83 (49.4%)	55 (41.0%)	28 (82.4%)	<0.001 ***	38 (36.9%)	17 (54.8%)	0.07
HPV vaccination is given as a series of either two or three doses, depending on the child’s age at initial vaccination. (T)	55 (32.7%)	33 (24.6%)	22 (64.7%)	<0.001 ***	22 (21.4%)	11 (35.5%)	0.11
The HPV vaccine is most effective when given to people who’ve never had sex. (T)	106 (63.1%)	79 (59.0%)	27 (79.4%)	0.03 *	61 (59.2%)	18 (58.1%)	0.91
Someone who has had the HPV vaccine cannot develop cervical cancer. (F)	80 (47.6%)	64 (47.8%)	16 (47.1%)	0.94	48 (46.6%)	16 (51.6%)	0.62
The HPV vaccine offers protection against most cervical cancers. (T)	55 (32.7%)	40 (29.9%)	15 (44.1%)	0.11	31 (30.1%)	9 (29.0%)	0.91
The HPV vaccine offers protection against genital warts. (T)	97 (57.7%)	73 (54.5%)	24 (70.6%)	0.09	55 (53.4%)	18 (58.1%)	0.65
Girls who have had a HPV vaccine do not need a Pap test when they are older. (F)	123 (73.2%)	94 (70.2%)	29 (85.3%)	0.07	69 (67.0%)	25 (80.7%)	0.15
The HPV vaccine protects you from every type of HPV. (F)	68 (40.5%)	53 (39.6%)	15 (44.1%)	0.63	39 (37.9%)	14 (45.2%)	0.47
HPV vaccination prevents new HPV infections but does not treat existing HPV infections or diseases. (T)	101 (60.1%)	76 (56.7%)	25 (73.5%)	0.07	56 (54.4%)	20 (64.5%)	0.32
You can cure HPV by getting the HPV vaccine. (F)	73 (43.5%)	52 (38.8%)	21 (61.8%)	0.02 *	36 (35.0%)	16 (51.6%)	0.10
**HPV literacy mean score (range 0–1) and SD**	0.50 (0.23)	0.46 (0.22)	0.66 (0.19)	<0.001 ***	0.44 (0.21)	0.52 (0.24)	0.05 *

Note: SD = standard deviation; *p* < 0.05 *, *p* < 0.001 ***.

**Table 4:** Bivariate associations between predisposing, enabling, and need factors and HPV vaccine initiation (N = 168) and vaccine intention (N = 134).

Variables	HPV Vaccine Initiation (N = 168)		HPV Vaccination Intention Within the Next 12 Months (N = 134)	
	n (%)	*p*-Value	n (%)	*p*-Value
**Predisposing Factors**				
**Parent age**	1.02 (0.99, 1.06)	0.19	0.95 (0.91, 0.99)	0.02 *
**Parent gender**				
Mother	2.55 (1.13, 5.74)	0.02 *	2.82 (1.21, 6.58)	0.02 *
Father	Ref		Ref	
**Parent marital status**				
Never married or other	Ref	0.82	Ref	0.76
Married or partnered	0.91 (0.39, 2.14)		1.16 (0.45, 3.00)	
**Parent ethnicity**				
Salvadoran	Ref		Ref	
Honduran	0.62 (0.24, 1.63)	0.33	1.40 (0.56, 3.48)	0.47
Guatemalan	0.98 (0.41, 2.35)	0.96	0.41 (0.13, 1.27)	0.12
**Parent length of residence in the U.S.**	1.05 (1.01, 1.09)	0.02 *	0.95 (0.91, 1.00)	0.03 *
**Primary language spoken at home**				
English	4.21 (0.81, 22.0)	0.09	0.00 (0.00, I)	0.98
Spanish	Ref		Ref	
Both	0.97 (0.26, 3.65)	0.97	0.96 (0.25, 3.75)	0.96
**Sex of the index child**				
Male	Ref	0.05	Ref	0.81
Female	2.13 (0.99, 4.56)		0.90 (0.39, 2.08)	
**Age of the index child**	1.19 (0.99, 1.44)	0.06	0.94 (0.77, 1.13)	0.50
**Index child birth place**				
Born outside of the U.S.	Ref	0.01 *	Ref	0.99
Born in the U.S.	3.35 (1.30, 8.63)		0.99 (0.44, 2.24)	
**Enabling Factors**				
**Educational level**				
<High school	Ref	0.36	Ref	0.16
≥High school	1.43 (0.67, 3.04)		1.78 (0.79, 4.02)	
**Household income**				
<USD $50,000	Ref	0.72	Ref	0.47
≥USD $50,000	1.16 (0.52, 2.57)		0.74 (0.32, 1.68)	
**Employment status**				
Working (yes)	3.14 (0.70, 14.08)	0.13	0.45 (0.17, 1.21)	0.11
Unemployed (no)	Ref		Ref	
**Received information about the HPV vaccine from a HCP (doctor or nurse)**				
Yes	62.00 (17.23, 223.15)	<0.001 ***	4.92 (1.78, 13.61)	0.002 **
No	Ref		Ref	
**HPV vaccine literacy score**				
**(Mean, SD)**	159.83 (14.32, 1783.79)	<0.001 ***	6.15 (0.83, 45.78)	0.08
**Need Factors**				
**Health insurance status**				
No	Ref	0.21	Ref	0.82
Yes	2.64 (0.58, 11.95)		1.15 (0.35, 3.76)	
**Family history of cervical cancer or HPV-associated cancers**				
No	Ref	0.26	Ref	0.29
Yes	1.93 (0.62, 5.98)		2.03 (0.55, 7.46)	
**Index child has regular HCP^1^**				
No	Ref	-	Ref	0.37
Yes	-		0.44 (0.07, 2.73)	
**HCP recommended the HPV vaccine^1^**				
No	Ref		Ref	<0.001 ***
Yes	-		13.64 (3.41, 54.52)	

Note: 1. Analysis of the HPV vaccine initiation and vaccination intention variables in the last two rows could not be conducted due to the presence of zero values in certain cells, which made it impossible to compute valid *p*-values or derive meaningful results for these categories. Additional data on these variables are provided in the Supplementary Materials; HCP = healthcare provider; *p* < 0.05 *, *p* < 0.01 **, *p* < 0.001 ***.

**Table 5:** Multiple logistic regression analysis: HPV vaccine initiation (n = 168) and intention to receive the HPV vaccine within the next 12 months (N =134).

Factors	HPV Vaccine Initiation (N = 168)		HPV Vaccination IntentionWithin Next 12 Months (N = 134)	
	OR	(95% CI)	OR	(95% CI)
**Predisposing factors**				
Sex child (female)	1.79	(0.43, 7.48)	1.27	0.37, 4.34
Age child	1.09	(0.80, 1.49)	1.15	0.85, 1.56
Country of birth of the index child (U.S.)	2.72	(0.40, 18.48)	10.47 *	1.51, 72.68
Parent age	0.98	(0.89, 1.09)	0.96	0.90, 1.03
Parent gender (mother)	0.42	(0.06, 3.04)	0.91	0.22, 3.82
Parent marital status (married)	0.78	(0.13, 4.52)	2.33	0.54, 10.05
Parent country of birth				
Salvadoran (Ref)	Ref	-	Ref	-
Honduran	0.77	(0.14, 4.16)	2.27	0.58, 8.93
Guatemalan	0.85	(0.16, 4.59)	0.58	0.11, 3.15
**Enabling factors**				
Educational level (≥ high school)	1.04	(0.27, 4.00)	1.42	0.40, 5.02
Household income (≥ USD $50,000)	2.61	(0.52, 13.04)	2.22	0.52, 9.44
Employment status (working)	3.00	(0.30, 30.26)	0.58	0.13, 2.56
Parent length of residence in the U.S.	1.03	(0.91, 1.16)	0.84 **	0.75, 0.95
Received information about the HPV vaccine from a HCP (doctor or nurse) (yes)	93.23 ***	(14.50, 599.63)	5.61	0.94, 33.30
HPV vaccine literacy score	25.14	(0.90, 703.66)	0.32	(0.02, 6.15)
**Need factors**				
Health insurance status (yes)	3.95	(0.26, 60.11)	3.56	0.68, 18.51
Family history of cervical cancer or HPV-associated cancers (yes)	2.95	(0.39, 22.14)	5.75	0.88, 37.62
HCP recommended the HPV vaccine to the child (yes)	--	--	14.73 *	1.77, 122.32

Note: HCP = healthcare provider; *p* < 0.05 *, *p* < 0.01 **, *p* < 0.001 ***.

Among the enabling factors, receiving information about the HPV vaccine from a HCP and vaccine literacy were the only two factors significantly associated with vaccine initiation. Parents who received information about the HPV vaccine from a HCP were 62 times more likely to initiate vaccination for their child, with an odds ratio of 62.00 (*p* < 0.001). Similarly, parents with higher HPV vaccine literacy, defined as a better understanding of the vaccine and its benefits, were significantly more likely to initiate vaccination (OR = 159.83, *p* < 0.001). None of the need factors, including health insurance status or a family history of cervical cancer or HPV-associated cancers, were significantly associated with vaccine initiation. All the children who had initiated the HPV vaccine had regular HCPs and had received the provider’s recommendation for the vaccine (See Supplementary Materials); therefore, they are not included in the results presented in Table 4.

In regard to the multiple logistic regression analysis, receiving information about the HPV vaccine from a HCP was the most significant factor for vaccine initiation (Table 5). Parents who received this information were approximately 93 times more likely to have initiated vaccination, controlling for all other variables, with an adjusted odds ratio (AOR) of 93.23 (95% CI = 14.50–599.63; *p* < 0.001).

None of the other factors showed a statistically significance association with vaccine initiation in the adjusted analysis. Due to high collinearity with receiving information from a HCP about the HPV vaccine, the HCP recommendation variable was not included in the adjusted model for vaccine initiation.

#### 3.4.2. Vaccine Intention Among Parents of Unvaccinated Children

The bivariate analysis for HPV vaccination intention within the next 12 months identified several significant associations between predisposing, enabling, and need factors (Table 4). Among the predisposing factors, several parental characteristics were associated with vaccine intention. Similar to vaccine initiation, the parent’s gender played a significant role in vaccine intention. Mothers were significantly more likely than fathers to intend to vaccinate their child (OR = 2.82, *p* = 0.02). The parent’s age was negatively associated with vaccination intention. Specifically, for each additional year in the parent’s age, the odds of intending to vaccinate decreased slightly (OR = 0.95, *p* = 0.02). Additionally, the parent’s length of residence in the U.S. was significantly associated with vaccine intention, with parents who had lived in the U.S. longer having slightly lower odds of intending to vaccinate their child within the next 12 months (OR = 0.95, *p* = 0.03).

No significant associations were found in regard to the parent’s marital status, the parent’s ethnicity, or language spoken at home, suggesting that these factors did not influence vaccine intention. Additionally, none of the child’s characteristics (sex, age, country of birth) were significantly associated with vaccine intention.

In terms of the enabling factors, receiving information about the HPV vaccine from a HCP was also strongly associated with a higher likelihood of intending to vaccinate within the next 12 months. Parents who reported receiving information about the HPV vaccine from a HCP had significantly higher odds of intending to vaccinate their child (OR = 4.92, *p* = 0.002). None of the other enabling factors (the parent’s educational attainment, employment status, household income, and HPV vaccine literacy) were significantly associated with vaccine intention.

Among the need factors, only receiving a recommendation from a HCP for the HPV vaccine was significantly associated with higher odds of intending to vaccinate. Parents who received a recommendation from a HCP were 13.64 times more likely to express the intention to vaccinate their child compared to those who did not receive such a recommendation (OR = 13.64, *p* < 0.001). None of the other need factors (health insurance, family history of cervical cancer or other HPV-associated cancers, and child having a regular HCP) were significantly associated with vaccine intention.

In the multiple logistic regression analysis, three factors predicted the parents’ intention to vaccinate their children within the next 12 months (Table 5). First, in the adjusted analysis, the parent’s length of residence in the U.S. was inversely related to vaccination intention, with parents who had lived longer in the U.S. less likely to intend to vaccinate their child (AOR = 0.84, 95% CI: 0.75, 0.95, *p* < 0.01) compared to those who had lived in the U.S. for a shorter duration, showing that each additional year of residence decreased the odds of intending to vaccinate by 16%. Second, after controlling for all the other variables, having a child born in the U.S. significantly increased the likelihood of intending to vaccinate (OR = 10.47, 95% CI = 1.51–72.68, *p* < 0.05), indicating that parents of U.S.-born children were approximately 10 times more likely to intend to vaccinate compared to parents of children born outside the U.S. The third significant predictor was receiving a recommendation from a HCP (doctor or nurse), which was strongly associated with vaccination intention (AOR = 14.73, 95% CI: 1.77, 122.32, *p* < 0.05), after adjusting for all the other factors. Parents who reported that a HCP had recommended the HPV vaccine were almost 15 times more likely to intend to vaccinate their child within the next 12 months compared to those who did not.

## 4. Discussion

This study aimed to examine factors influencing HPV vaccination behaviors (initiation and intention) among Central American parents of adolescents in the U.S., specifically those from the Northern Triangle countries, El Salvador, Guatemala, and Honduras. To our knowledge, it is one of the first studies to focus specifically on this demographic, providing valuable insights for tailored public health strategies to increase vaccine uptake in this group [7,8,9]. The results highlight key factors related to sociodemographic, HPV vaccine knowledge, and the critical role of HCPs in shaping vaccination behaviors. Our findings emphasize the significant impact of HCP recommendations and parental understanding of the HPV vaccine on both vaccine initiation and the intention to vaccinate.

### 4.1. Vaccine Initiation and Intention Rates

Our findings revealed that only approximately 20% of the children in the sample had initiated the HPV vaccination. Furthermore, among the parents of unvaccinated children, only about 23% expressed an intention to vaccinate their child within the next 12 months. These rates are notably lower than those reported in previous studies of Latino parents and other ethnic groups [7,8], but are comparable to some other studies [50,51]. For instance, a 2020 study examining HPV vaccine coverage across Hispanic/Latinx subgroups, using data from the 2012–2016 National Immunization Survey-Teen (n = 16,335) on Hispanic/Latinx adolescents aged 13–17, found initiation rates among females ranging from 63.4% among Cubans to 71.2% among Puerto Ricans, with completion rates ranging from 33.6% among multi-subgroup females to 48.7% among South Americans [7]. In contrast, the same study reported that vaccine coverage among males was highest among Central Americans, with an initiation rate of 57.5% [7].

In contrast to these higher rates, a 2013 study of Latina mothers from a Federally Qualified Health Center serving low-income families in Florida found that only 18% of their daughters had received at least one dose of the HPV vaccine [50]. Similarly, a more recent study (2018), among low-income Hispanic females aged 11–17 years, found that only 21% had initiated the HPV vaccine series [51]. These rates are similar to the 20% initiation rate found in our study, further suggesting that low vaccine uptake may be a common issue among underserved Latino populations. Moreover, the Florida study also found that higher levels of U.S. acculturation were associated with greater odds of vaccine uptake [50]. Similarly, the lower vaccine initiation rate in our study may, in part, be explained by the fact that nearly all the parents in our sample were immigrants with low acculturation levels (94.6% had a SASH score < 2.99), a factor that may contribute to reduced vaccine uptake by this population.

### 4.2. Sociodemographic Factors and Vaccine Behavior

Our findings indicate significant gender differences in regard to HPV vaccine initiation and the intention to vaccinate, with mothers reporting higher rates of both vaccination and the intent to vaccinate compared to fathers. This aligns with previous research suggesting that mothers are typically more involved in healthcare decisions for their children, particularly in Latino families [52,53,54]. The disparity in vaccine initiation between mothers and fathers may also reflect differing levels of exposure to health information and engagement with HCPs, as the mothers in our sample were more likely to report receiving information from a HCP. This highlights the need for targeted health communication strategies that engage fathers more actively in discussions about vaccines.

Additionally, the length of time parents had lived in the U.S. was positively associated with both vaccine initiation and the intention to vaccinate. Parents with longer U.S. residency were more likely to initiate the HPV vaccine, possibly reflecting greater acculturation and increased familiarity with U.S. healthcare practices. However, in the multivariate analysis, longer U.S. residency was inversely associated with vaccine intention, suggesting that while acculturation may promote vaccine initiation, it could also normalize lower vaccine uptake over time within certain immigrant populations. Previous studies have highlighted the importance of considering acculturation in HPV vaccination decisions [14,26,50]. Together with our findings, these studies point to the need for further research to explore the complex dynamics of acculturation and how long-term immigrant parents’ perceptions of U.S. healthcare might evolve.

In terms of the child’s characteristics, U.S.-born children were significantly more likely to have received the HPV vaccine compared to those born outside the U.S., a finding that is consistent with studies showing that U.S.-born children of immigrant parents often have better access to healthcare services and are more likely to receive recommended vaccinations [11,12,14,15]. This highlights the need for interventions that address vaccination disparities among immigrant families, particularly those with children born outside the U.S.

### 4.3. Role of Healthcare Providers and Vaccine Literacy

A key finding in our study is the critical role of HCP communication and recommendations in influencing both HPV vaccine initiation and the intention to vaccinate. Parents who received information from a HCP were significantly more likely to initiate vaccination for their child, with an odds ratio of 93.23. Furthermore, after adjusting for other factors, receiving information about the HPV vaccine from a HCP remained a strong predictor of vaccine intention. Parents who received such recommendations had nearly 15 times higher odds of intending to vaccinate their child within the next year. These findings align with previous research and emphasize the essential role of HCP engagement in shaping vaccination decisions, especially for parents who have not yet initiated vaccination [36,40,41,55,56,57,58].

Consistent with the extensive literature on the matter, our results confirm that HCPs play a pivotal role in vaccine decision making, particularly in underserved populations [36,40,41]. This highlights the importance of ensuring that HCPs proactively discuss the HPV vaccine with Central American immigrant parents, who may be less familiar with vaccine guidelines, or the cancer risks associated with HPV [53]. In fact, after adjusting for other factors, receiving information from a HCP remained a significant predictor of vaccine intention (AOR = 14.73, 95% CI: 1.77, 122.32, *p* < 0.05), underscoring the ongoing importance of HCP engagement in promoting vaccine uptake [40,41,55,56,57,58].

In addition to the role of HCP recommendations, our analysis of HPV vaccine literacy revealed that parents who had initiated the HPV vaccine for their child had significantly higher vaccine literacy scores. This suggests that improving parental knowledge about the vaccine and its benefits could be an effective strategy for increasing vaccine uptake. However, the low overall vaccine literacy in our sample, with only 50% of parents correctly answering at least half of the HPV vaccine literacy questions, highlights the need for targeted educational interventions to improve the understanding of the vaccine’s role in preventing HPV-related cancers. Educational campaigns should address common misconceptions, such as the belief that the HPV vaccine protects against all sexually transmitted infections or can cure existing HPV infections [11,15,17,18,19,20,24,34,40,42,44,45,59].

It is important to note that while vaccine literacy was associated with higher rates of vaccine initiation in our unadjusted analysis, this relationship did not persist after adjusting for other factors. Specifically, once we accounted for the strong influence of HCP recommendations, the association between vaccine literacy and initiation was no longer significant. This suggests that while parental vaccine literacy influences vaccine decisions, the direct impact of HCP recommendations may be more important in determining vaccine behaviors [17,18,19,59]. These findings underscore the need to integrate educational efforts with provider-based interventions to maximize vaccine uptake.

### 4.4. Barriers to Vaccine Initiation and the Intention to Vaccinate

Our study found that only 20% of parents reported that their child had initiated the HPV vaccine and fewer than a quarter expressed an intention to vaccinate within the next 12 months, despite limited HCP interaction. These low vaccination rates may be attributed to various factors, including cultural beliefs, mistrust of the healthcare system, and concerns about vaccine safety [9,14,15,16,17,23,24]. Notably, while HCP recommendations were strongly associated with both vaccine initiation and the intention to vaccinate, many parents reported low levels of HCP recommendations, with fewer than 28% indicating that their child’s HCP had recommended the HPV vaccine. This highlights the need for targeted interventions to encourage HCPs to more actively recommend the vaccine, especially for Latino families who may face language barriers or cultural differences that impact vaccine discussions [17,18,19,40,42,44,45].

In terms of need factors, neither health insurance status nor a family history of cervical cancer was significantly associated with vaccine initiation in the final adjusted multiple logistic regression model. This suggests that while having health insurance may improve access to healthcare services, these factors alone are not sufficient to drive vaccination behavior. Instead, the only significant factor remaining, associated with vaccine initiation, was receiving information about the HPV vaccine from a HCP. This finding underscores the critical role of HCP engagement in motivating vaccination, even in the presence of other health-related factors [7,8,31,51,52,53].

In terms of vaccine intention, three factors remained significantly associated in the final adjusted model: the child’s country of birth (with U.S.-born children showing higher odds of vaccination), the parent’s length of residence in the U.S., and receiving a HCP recommendation. These findings emphasize the complex interplay of factors influencing vaccine intentions, suggesting that both demographic factors and HCP engagement are crucial in shaping vaccine decisions among Latino families [8,58,59,60,61,62,63,64].

### 4.5. Implications for Public Health Interventions

Our findings suggest several important strategies for improving HPV vaccine uptake among Latino families. First, HCPs must be equipped with the knowledge and tools to discuss the HPV vaccine with parents, particularly in settings with high percentages of Latino immigrants. Training providers to offer clear, culturally sensitive, and language-appropriate information about the vaccine could help address parental concerns and increase vaccination rates. Second, given the important role of parental vaccine literacy, educational campaigns should focus on improving parents’ understanding of the HPV vaccine and dispelling myths surrounding its efficacy and safety. These campaigns should leverage trusted sources of information, such as HCPs, as well as community-based resources to engage both mothers and fathers in the vaccination conversation [51,58,59,60,61,62,63,64].

Finally, interventions should address the gender disparities observed in vaccine initiation and intention to vaccinate by targeting fathers more directly [65]. By providing fathers with accurate, accessible, and culturally relevant information about the HPV vaccine, public health campaigns could help close the gap in vaccine uptake between mothers and fathers [65,66].

### 4.6. Limitations and Future Research

The findings should be interpreted with several limitations in mind. First, the cross-sectional design limits causal inferences [67] and selection bias may arise due to the voluntary nature of survey participation. Additionally, the relatively small sample size (N = 168) may have constrained the study’s power to detect significant associations between HPV vaccine literacy and vaccination behaviors. The study was conducted with a convenience sample of Central American parents, the majority of which were immigrants with low acculturation levels, which limits the generalizability of our findings to other ethnic or racial groups [67]. Self-reported data could also be affected by recall bias, particularly about HCP’s HPV vaccine recommendations. Finally, the study relied on self-reported data, which may be subject to recall bias or social desirability bias, particularly regarding vaccine uptake and intention [68].

Despite these limitations, this study provides valuable insights into Central American immigrant parents, an underrepresented group, and highlights the unique challenges and barriers they face. A key strength of the study is its inclusion of both mothers and fathers, offering a more comprehensive perspective. Additionally, the use of an interviewer-administered survey by bilingual native Spanish speakers minimizes misunderstandings and ensures cultural and linguistic relevance, as most participants reported Spanish as their primary language at home [69,70]. By including both mothers and fathers, our study contributes to the literature by addressing a gap that stems from the fact that most studies have focused solely on mothers, thus broadening the understanding of Central American immigrant parents’ viewpoints on HPV vaccination [65,66].

## 5. Conclusions

In conclusion, our study highlights the significant influence of HCP recommendations and vaccine literacy on both HPV vaccine initiation and the intention to vaccinate among Central American immigrant parents from El Salvador, Guatemala, and Honduras. The findings underscore the critical role that HCPs play in shaping vaccination decisions, particularly in underserved populations. Parents who received information from a HCP were far more likely to initiate vaccination and express an intention to vaccinate their children in the future. This underscores the need for proactive HCP engagement, especially in communities with low vaccine literacy, to improve HPV vaccine uptake and reduce disparities in HPV-related cancer prevention.

Our findings also emphasize the importance of addressing vaccine knowledge gaps. Given the relatively low levels of vaccine literacy observed in our study, targeted educational interventions focused on increasing parental understanding of the HPV vaccine and its benefits could be an effective strategy for improving vaccine initiation and the intention to vaccinate. Tailored communication efforts, designed to engage both mothers and fathers, are particularly crucial within Central American immigrant communities, where cultural nuances and language barriers may affect health-related decision making.

Furthermore, our study highlights significant sociodemographic factors, including gender disparities and the child’s country of birth, that should be considered when designing public health campaigns. The moderate level of vaccine literacy and differences in vaccination rates observed across demographic groups emphasize the need for culturally sensitive approaches that consider the unique needs of immigrant populations. Enhancing HCP training, developing tailored communication strategies, and improving provider–patient interactions are essential steps toward boosting HPV vaccine uptake.

Public health efforts should prioritize increasing HPV vaccine literacy through culturally relevant educational programs, community outreach, and accessible resources. Future research, particularly using longitudinal data, should explore the cultural and contextual factors that influence vaccine decision making within Latino communities, as well as the impact of interventions aimed at improving vaccine knowledge and provider recommendations. These efforts will be crucial in addressing the barriers to vaccination and achieving higher vaccination rates among immigrant populations.

## Data Availability

Due to privacy or ethical restrictions, the data that support the findings of this analysis are not publicly available; however, they are available upon request from the corresponding author.

## Supplementary Materials

The following supporting information can be downloaded at: https://www.mdpi.com/article/10.3390/vaccines13020130/s1; Table S1: Predisposing, enabling and needed factors by HPV vaccine initiation (N = 168) and vaccine intention (N = 134).
