# Supplementary material for: Understanding HPV Vaccine Initiation and Intention Among Central American Immigrant Parents in the United States: The Role of Vaccine Literacy and Healthcare Provider Recommendations
Source: Vaccines (Basel). 2025 Jan 27;13(2):130. doi: 10.3390/vaccines13020130 (PMC11860528; doi:10.3390/vaccines13020130)
Supplement: Supplementary file 1 [file vaccines-13-00130-s001.zip › vaccines-3406075-supplementary.pdf]

Table S1. Predisposing, enabling and needed factors by HPV vaccine initiation (N = 168) and vaccine intention (N=134).

Variables	Total Sample N (%)	HPV Vaccine Initiation (N=168)		HPV Vaccination Intention Within the Next 12 months (N=134)	
		n (%)	*p*-value	n (%)	*p*-value
PREDISPOSING FACTORS					
**Parent age**	45.3 (10.1)	47.3 (7.3)	0.05*	40.9 (7.3)	0.03*
**Parent gender**					
Mother	89 (53.0%)	24 (70.6%)	0.02*	21 (67.7%)	0.01*
Father	79 (47.0%)	10 (29.4%)		10 (32.3%)	
**Parent marital status**					
Never married or other	42 (25.0%)	9 (26.5%)	0.82	7 (22.6%)	0.76
Married or partnered	126 (75.0%)	25 (73.5%)		24 (77.4%)	
**Parent ethnicity**					
Salvadoran	62 (36.9%)	14 (41.2%)	0.58	12 (38.7%)	0.08
Honduran	52 (31.0%)	8 (23.5%)		14 (45.2%)	
Guatemalan	54 (32.1%)	12 (35.3%)		5 (16.1%)	
**Parent length of residence in the U.S.**	16.9 (9.6)	20.4 (8.3)	0.03*	12.7 (8.7)	0.03*
**Primary language spoken at home**					
English	6 (3.6%)	3 (8.8%)	0.17	0 (0.0%)	1.00
Spanish	146 (86.9%)	28 (82.4%)		28 (90.3%)	
Both	16 (9.5%)	3 (8.8%)		3 (9.7%)	
**Sex index child**					
Male	99 (58.9%)	15 (44.1%)	0.05*	20 (64.5%)	0.81
Female	69 (41.1%)	19 (55.9%)		11 (35.5%)	
**Age index child**	14.2 (2.2)	14.8 (2.24)	0.05*	13.8 (2.3)	0.42
**Country of birth index child**					
Outside of the U.S.	62 (36.9%)	6 (17.6%)	0.01**	13 (41.9%)	0.99
In the U.S.	106 (63.1%)	28 (82.4%)		18 (58.1%)	
ENABLING FACTORS					
**Educational level**					
< High school	86 (51.2%)	15 (44.1%)	0.36	13 (41.9%)	0.16
≥ High school	82 (48.8%)	19 (55.9%)		18 (58.1%)	

**Household income**					
< $50,000	64 (40.3%)	12 (37.5%)	0.72	14 (46.7%)	0.47
≥ $50,000	95 (59.8%)	20 (62.5%)		16 (53.3%)	
**Employment status**					
Working (yes)	144 (85.7%)	32 (94.1%)	0.07	23 (74.2%)	0.11
Unemployed (no)	24 (14.3%)	2 (5.9%)		8 (25.8%)	
**Received information about the HPV vaccine from an HCP (doctor or nurse)**					
Yes	50 (29.9%)	31 (91.2%)	< 0.001***	10 (32.3%)	< 0.001***
No	117 (70.1%)	3 (8.8%)		21 (67.7%)	
**HPV Vaccine Literacy Score**					
**(Mean, SD)**	0.5 (0.2)	0.7 (0.2)	< 0.001***	0.5 (0.2)	0.05*
NEED FACTORS					
**Health insurance status**					
No	21 (12.5%)	2 (5.9%)	0.25	4 (12.9%)	1.00
Yes	147 (87.5%)	32 (94.1%)		27 (87.1%)	
**Family history of cervical cancer or HPV-associated cancers**					
No	152 (90.5%)	29 (85.3%)	0.25	27 (87.1%)	0.28
Yes	16 (9.5%)	5 (14.7%)		4 (12.9%)	
**Child having regular HCP**					
No	5 (3.0%)	0 (0.0%)	0.58	2 (6.5%)	0.33
Yes	163 (97.0%)	34 (100.0%)		29 (93.6%)	
**HCP recommendation of the HPV vaccine**					
No	122 (72.6%)	0 (0.0%)	< 0.001***	22 (71.0%)	< 0.001***
Yes	46 (27.4%)	34 (100.0%)		9 (29.0%)	

$p \leq 0.05$*, $p < 0.01$**, $p < 0.001$***
